# Substitution and Imitation Again

**Published:** 1902-07

**Authors:** 


### Substitution and Imitation Again.

ALMOST at the time we were writing an article relating to this subject that appeared in our edition for June, a trial was going on in New York that resulted in the conviction and fine of a druggist for substituting another preparation in place of the one prescribed. Messrs. Fairchild Brothers & Foster, the well-known manufacturing chemists, a house whose integrity is unquestioned, for some time past have been aware that imitation mixtures have been dispensed in place of their essence of pepsin. They have instituted prosecution in several instances, and the result in one of these is thus told in the New York press of May 24, 1902:

In Special Sessions on last Tuesday before Justices Wyatt, McKean and Hinsdale, Clarence D. Bowman, a director of the Lewis A. Bates Company and the manager of their drug store in No. 739 Sixth avenue, pleaded guilty to having violated section No. 364 of the penal code in using another preparation in place of essence of pepsin manufactured by Fairchild Brothers & Foster, in filling prescriptions calling for the latter preparation. He was fined $50.

It appeared that on several occasions when physicians had prescribed Fairchild's pepsin Bowman had delivered the imitation mixture. Bowman said he was sorry for what he had done, but had no excuse to offer. In imposing sentence Justice Wyatt said that the offense was a most serious one, and that a heavier penalty would have been imposed had not the injured firm recommended leniency by reason of its being the defendant's first conviction.

We are pleased to record this additional conviction for a crime that ought to be punished with all the severity the law permits. It is a subject in which physicians take a deep interest and they should unite with druggists and pharmacists in bringing all such offenders to the bar of justice.

---

THE Buffalo Society for the Prevention of Tuberculosis is to be congratulated upon the good service it rendered the citizens of Buffalo through a public meeting held at the Central Presbyterian Church on the evening of May 30, 1902. Dr. Herman M. Biggs, medical officer of the New York board of health, who may be regarded as a specialist in the prevention and management of tuberculosis, addressed a large audience on consumption and its prevention. During his discourse Dr. Biggs made many telling points and especially emphasised the fact that pulverised dry sputum was the chief source of communicating the disease from man to man.

The society recently elected the following-named officers for the current year: president, Dr. Benjamin G. Long; vice-president, Dr. Henry R. Hopkins; treasurer, Dr. A. H. Briggs; secretary, Dr. William G. Bissell.

---

Dr. George Ryerson Fowler, of Brooklyn, has recently instituted a series of suits to collect medical fees from patients who go to hospitals for the purpose of securing gratuitous services in surgical operations, though able to pay for the same. It is a practice that should be broken up and Dr. Fowler is to be congratulated upon the valuable services he is rendering hospitals, physicians and the general public in the premises. Thus far all the suits have been decided in his favor. Recently another has been disposed of before Justice Van Wart, in the IId District Municipal Court, who awarded to William E. Taylor $160 for professional services rendered to Julius Anderson and his fourteen-year-old daughter. Dr. Fowler assigned his claim to Mr. Taylor. An operation for cancer of the lower jaw was performed upon Anderson by Dr. Fowler. The former claimed that as he had paid $7 a week for treatment in the Seney Hospital, where the operation was performed, no additional charges could be brought against him. Medical services were rendered to Miss Anderson.

Justice Van Wart allowed the claim on the ground that "when one accepts a beneficial service from another the law presumes an intention on his part to pay therefor the reasonable value of the services rendered, and an agreement to that effect is implied."

---

The Buffalo Fresh Air Mission has begun its summer campaign with its usual energy. One of its special provinces is to foster the fresh air mission hospital at Athol Springs where many improvements have been planned for the coming season. The subject of enlarging the hospital is under consideration. The present capacity is 100, and last year there was a suggestion of overcrowding.

Mr. Jacob A. Riis, of New York, recently gave a lecture which has stimulated interest in the work, and the officers feel that there was never a more opportune time than now for raising funds for any branch of the Fresh Air Mission.

The newly elected officers of the hospital are: president, Frederick Truscott; vice-presidents, Paul C. Ransom, Dr. D. W. Harrington; treasurer, Henry W. Sprague; secretary, John R. Williams; general manager, Frederic Almy; manager, Marc W. Comstock; cradle bank manager, W. H. Wright, Jr.; medical staff, Irving M. Snow, Dewitt H. Sherman, Albert T. Lytle, Eugene A. Smith, Nelson G. Russell and Lorenzo Burrows, Jr.

---

DR. GEORGE MILLER STERNBERG, surgeon-general U. S. Army (retired), was tendered a dinner at Delmonico's, New York, Friday evening, June 13, 1902, by about 100 prominent physicians and surgeons from various portions of the country. Dr. E. G. Janeway, of New York, presided and felicitous speeches were made by several of the gentlemen present commendatory of General Sternberg's military and scientific professional work. Dr. Sternberg's rare accomplishments as well as his courageous service in two wars were dwelt upon and the occasion was one long to be remembered by this able, efficient and brave officer. It is to be regretted that the operation of the law is imperative and removes from active service such a capable man while yet in the full vigor of manhood and plenitude of power.

